# High Throughput Laser Process of Transparent Conducting Surfaces for Terahertz Bandpass Ultrathin Metamaterials

**DOI:** 10.1038/s41598-019-38949-1

**Published:** 2019-02-28

**Authors:** Qinghua Wang, Michaella Raglione, Baojia Li, Xin Jin, Fatima Toor, Mark Arnold, Hongtao Ding

**Affiliations:** 1 Department of Mechanical Engineering, University of Iowa, Iowa City, Iowa 52242 USA; 2 Department of Chemistry, University of Iowa, Iowa City, Iowa 52242 USA; 3 School of Materials Science and Engineering, Jiangsu University, Zhenjiang, 212013 China; 4 Department of Electrical and Computer Engineering, University of Iowa, Iowa City, Iowa 52242 USA

## Abstract

Terahertz (THz) imaging has attracted much attention within the past decade as an emerging nondestructive evaluation technique. In this paper, we present a novel Laser-based Metamaterial Fabrication (LMF) process for high-throughput fabrication of transparent conducting surfaces on dielectric substrates such as glass, quartz and polymers to achieve tunable THz bandpass characteristics. The LMF process comprises two steps: (1) applying ultrathin-film metal deposition, with a typical thickness of 10 nm, on the dielectric substrate; (2) creating a ~100-micron feature pattern on the metal film using nanosecond pulsed laser ablation. Our results demonstrate the use of laser-textured ultra-thin film with newly integrated functional capabilities: (a) highly conductive with ~20 Ω/sq sheet resistance, (b) optically transparent with ~70% transmittance within visible spectrum, and (c) tunable bandpass filtering effect in the THz frequency range. A numerical analysis is performed to help determine the fundamental mechanism of THz bandpass filtering for the LMF-built samples. The scientific findings from this work render an economical and scalable manufacturing technique capable of treating large surface area for multi-functional metamaterials.

## Introduction

Imaging or sensing using terahertz (THz) electromagnetic radiation has emerged as a highly promising nondestructive evaluation technique^1,2^ for wide-ranging applications in agriculture and food science^3,4^, communication^5,6^, medical biology^7,8^, and security screening^9^. There is a growing need in the development of new THz devices to generate, detect and manipulate THz waves^10,11^. However, it remains a challenging task for scaling up the manufacturing process of current designs of THz devices, particularly those with an ease of tuning in the THz-domain. In addition, most of the available THz materials are usually not transparent within the visible spectrum, which makes them not utilizable for broad applications^12^.

Metallic THz filters on transparent substrates are practical and effective devices for filtering THz radiation. They can provide applications, such as THz frequency calibration artifacts and as a deterrent against counterfeiting^13^. The tunable and transparent THz metamaterial based multifunctional surface will be of vital importance for design and fabrication of THz bandpass filtering device with a glass-based substrate^14^. For example, there is a growing need to integrate THz optics or windows with electronic tuning or heating functions onto the windows of vehicles and airplanes for applications, such as camouflage and signal reception/filtering^15,16^. Meanwhile the visible transparency as a window coating and electrical conductivity for tuning THz response of the windows have to be maintained. It is therefore of great interest to incorporate these three surface functionalities into one single surface owing to the above-mentioned requirements and to develop a low-cost manufacturing solution.

Metamaterials, defined as engineered composites exhibiting properties that are not found in nature^17–19^, have been designed and developed for THz-domain applications^20^. A main thrust in THz-domain metamaterial design is transmission through sub-wavelength hole array structures. Cao and Nahata^21^ demonstrated the resonantly enhanced THz transmission through a periodic sub-wavelength hole array, which was perforated on free-standing stainless steel metal foils of 75 µm thickness. Significant enhancement of THz transmission was observed relating to the hole periodicity and the refractive index of the surface plasmon polariton (SPP) waves at the metal dielectric interface. Rivas *et al*.^22^ structured gratings on doped silicon and characterized the extraordinary THz transmission of the periodic structure. They attributed the phenomenon to the excitation of surface plasmon polariton on these structures and the subsequent tunneling through the periodic subwavelength holes. Matsui *et al*.^23^ discovered that in contrast to the conventional view, enhanced THz transmission resonances can be obtained by quasiperiodic (also termed aperiodic) hole structures in metal films. The lineshapes of these quasiperiodic surface structure have a width comparable with those of resonances obtained with periodic structures and could be used to achieve desirable transmission properties. Miyamaru *et al*.^24^ designed a surface-wave sensor to detect very small change in the substances by monitoring the transmission spectrum. The effects of design factors on THz transmission have been well studied, which includes hole periodicity^25^, hole shape^26,27^, hole orientation^28^, dielectric functions of metals^29^, hole size^30,31^, and thin dielectric layers^32^. Even with these recent research advancements, however, the state-of-the-art THz frequency selective filters still lack the frequency-domain modulating functionality, and often utilize metal foils with thickness of several tens of microns, which severely reduces their optical transmission in visible spectrum.

State-of-the-art fabrication methods of transparent conducting films utilizing metals include laser processing^33^, electrospinning^34^, inkjet printing^35^, photolithography^36,37^ and nanoimprinting^38^. Among these fabrication methods, direct laser fabrication of transparent conducting films has become a popular method due to its advantages in terms of good process flexibility and low manufacturing cost. Paeng *et al*.^39^ developed a nanosecond laser ablation method for flexible transparent conducting electrodes on copper (Cu), during which periodic metallic hole array structures were directly produced on different heat-sensitive flexible substrates without introducing significant thermal damage. The transparent conducting films prepared using the method proposed in [35] achieved a sheet resistance of 17.48 Ω/sq and a visible transmittance at 550 nm of 83%. The authors also demonstrated an improved photoelectric performance for their laser ablated transparent conducting film compared with commercial indium tin oxide (ITO) transparent conductive film. However, the laser ablation process throughput in their method was extremely low, which took more than four hours to process a 36 mm × 36 mm area, making it an unsuitable process for scaling up. A similar method developed by Lim *et al*.^40^ utilized ultrafast laser at a high pulse repetition rate to selectively remove self-assembled silver (Ag) nanoparticle network on polyethylene terephthalate (PET) substrate for fabrication of flexible and transparent film heaters. Besides laser ablation, other direct laser patterning methods such as selective laser sintering^33,41–44^, laser nano-welding^45^ and laser annealing/texturing^46–57^ were also used by researchers to fabricate transparent conducting films. To the best of our knowledge, THz transmission characteristics of transparent conducting films has not been investigated to-date utilizing laser textured metallic films.

**Figure 1:**
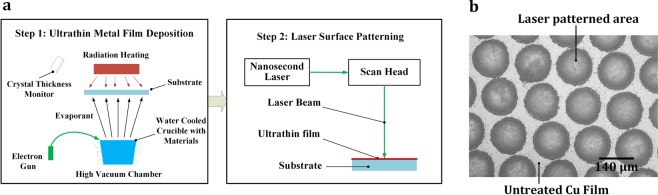
(**a**) Schematics of LMF experiments with Step 1 illustrating the ultra-thin metal film deposition process utilizing E-beam evaporator and Step 2 showing the laser surface patterning process; (**b**) optical image of a LMF surface patterned on an 8 nm-thick Cu film.

New materials processing science is needed to incorporate visible transparency and electrical conductivity into the design and fabrication of THz bandpass filters. In this paper, we present an innovative surface engineering method for dielectric material substrates such as glass, quartz and polymer, which is aimed to achieve the following integrated surface capabilities: (1) electrically conductive, (2) optically transparent, and (3) tunable bandpass filtering effect in the THz frequency range of 0.3 to 3 THz (or wavelength range of 100 µm to 1 mm). We name this proposed process: “Laser-based Metamaterial Fabrication (LMF)”, which comprises of two steps: (1) applying ultrathin-film metal deposition, with a typical thickness of 10 nm, on the dielectric substrate; (2) laser patterning of the coated surface using a Q-Switched Nd:YAG nanosecond pulsed laser (1,064 nm wavelength) with a typical feature size on the order of 100 µm. The schematic of LMF setup is shown in Fig. 1(a).

The results presented in this manuscript constitute the first attempt to achieve the combined functions of THz filtering effect, visible transparency, and electrical conductivity for dielectric substrate materials. Conventionally, metal mesh with a much higher thickness (tens of microns) is employed for THz devices/filters. In order to enhance the transparency of the THz bandpass filter, our method employs an ultra-thin metal film deposition. A fast one-step laser patterning process is developed to generate periodic surface patterns to make the THz bandpass filter fully functional. In comparison, the other existing fabrication methods are either too costly or time-consuming for periodic surface patterns used for THz bandpass filters^58^. Our research demonstrates for the first time that an ultrathin metal film (~10 nm in thickness) with periodic microscale patterns can have a bandpass filtering effect in the THz spectra. The fabricated transparent conducting THz bandpass filters can be used to select specific frequencies of interest and remove undesirable background radiation. The transparent conducting THz bandpass filter can be further used in devices, such as, signal receiver on the window of military vehicles^59^, electronic skin^60,61^ and human body sensor^62^.

## Results and Discussion

### Laser-based metamaterial fabrication

Various thin film deposition processes and materials have been experimentally evaluated in order to provide a viable fabrication solution of ultrathin-film metal deposition for LMF. Two different metals, Cu and aluminum (Al), and two different glass-based substrate materials, soda-lime glass (abbreviated as “glass”) with a thickness of 1 mm and quartz with a thickness of 500 µm, were used respectively as deposition and substrate materials. Two different film deposition methods were respectively used for Cu and Al. For coating of the substrate with Cu film, pure Cu was deposited using the Angstrom Engineering’s 6-pocket electron beam evaporator system. During the electron beam evaporation process, the thickness of the deposited metal was monitored *in-situ* by a piezoelectric thickness monitor. In this work, no binding agent was used on the substrate before the E-beam deposition process. This was deemed appropriate as the Cu film did not detach from the glass-based substrate even after a long time of storage and various tests. For coating the substrate with Al film, pure Al was deposited using the direct current (DC) magnetron sputtering technique. The deposition facility used was an IntlVac-Nanochrome I Sputterer. During the DC magnetron sputtering process, the power was set at 400 W and the Ar gas flow rate was 16.5 sccm. Using these two deposition techniques respectively, Cu and Al films with thickness of around 10 nm were deposited onto the substrates.

A 1,064 nm wavelength Q-Switched Nd:YAG nanosecond laser was selected for the second step of laser patterning. A 3-axis galvanometer laser scanner (SCANLAB intelliSCAN® 20 and varioSCANde 40i), configured with an f-theta objective, served to direct the laser beam with a beam size of ~100 µm onto the sample. The substrate materials of glass or quartz are highly transparent at this laser wavelength and hence absorbs little laser energy during patterning. It was determined that the long pulse mode of 120 ns duration with a pulse energy on the order of hundreds of mJ would be appropriate for laser patterning of the ultrathin film. The nanosecond laser ablation of Cu thin films enabled material removal without inducing thermal damage of the underlying glass or quartz substrate, allowing for the metallic micro-hole array to be directly produced on the substrate. An optical micrograph of the laser patterned micro-hole array pattern is shown in Fig. 1b. The current LMF process used a laser line spacing of ~150 µm and a processing speed of ~1.5 mm/s. It is noted that this work applied a high energy nanosecond laser with a repetition rate of 10 Hz. The processing speed can be substantially increased by adopting higher pulse repetition lasers. A 36 mm × 36 mm area could be fabricated within 6 s using a nanosecond laser with a typical 10 kHz repetition rate. The high throughput of our laser texturing technique will enable large-area processing for industrial applications.

### Sheet resistance

**Figure 2:**
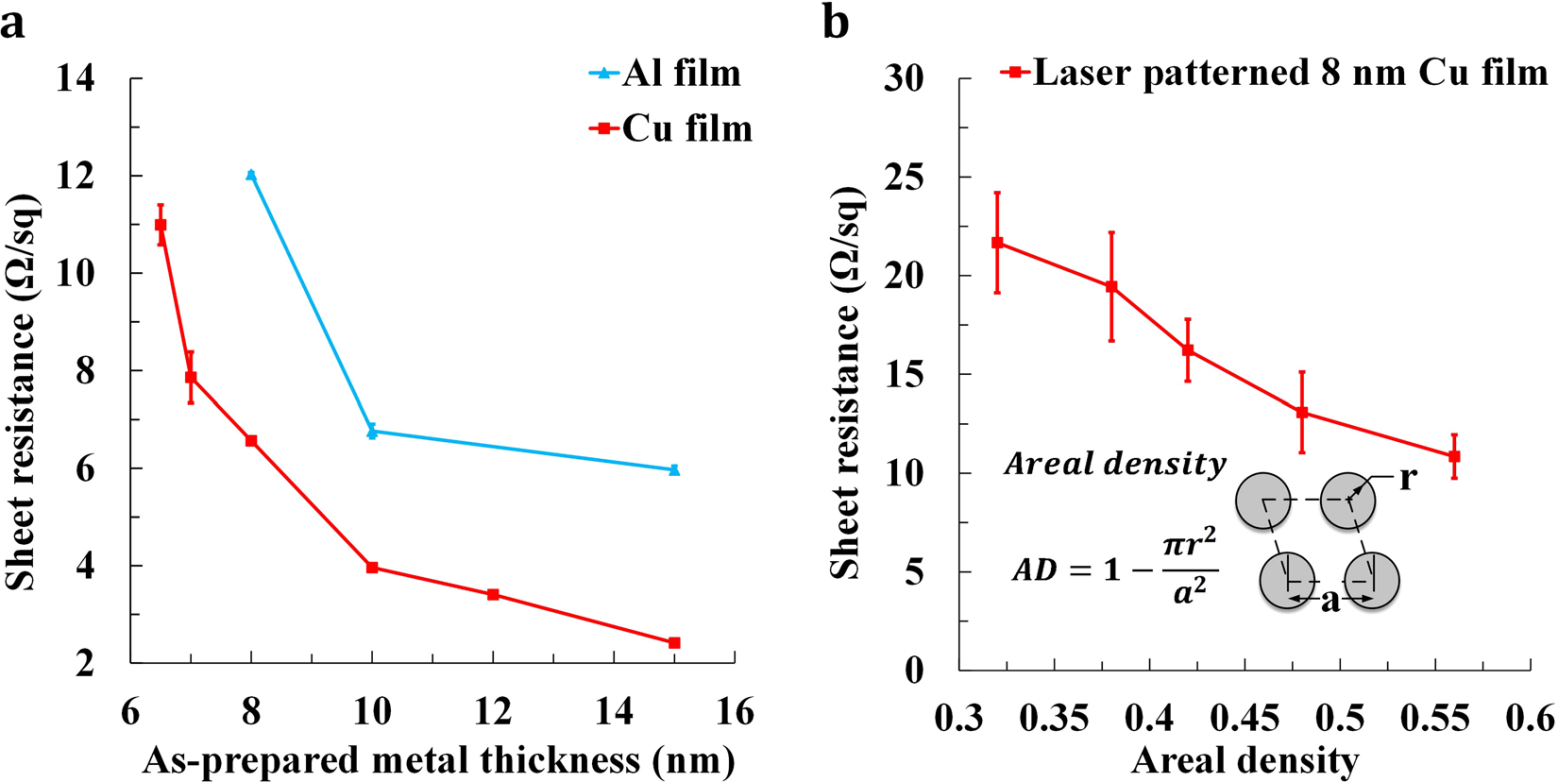
Sheet resistance of (**a**) as-deposited Cu and Al films as a function of layer thickness; (**b**) 8 nm-thick laser patterned Cu thin film as a function of areal density.

The sheet resistance was quantitatively measured to demonstrate the electrical conductivity of the LMF-built specimens. Four-point probe sheet resistivity measurements were initially performed for the as-deposited ultrathin Al and Cu films with varying thickness utilizing a Signatone Pro4 series system, as shown in Fig. 2a. For the as-deposited Al film, as the film thickness increased from 8 nm to 15 nm, the sheet resistance gradually decreased from 12.0 Ω/sq to 6.0 Ω/sq. For the as-deposited Cu film, as the film thickness increased from 6.5 nm to 15 nm, the sheet resistance gradually decreased from 11.0 Ω/sq to 2.4 Ω/sq. It can be found that the as-deposited Cu film has lower sheet resistance than the as-deposited Al film with same film thickness because Cu has much better electrical conductivity than Al.

The sheet resistance of the LMF-built samples varies with the laser pattern. A key parameter is areal density, which means the ratio of residual area after LMF process over the total area as illustrated in Fig. 2. As the areal density increased from 0.32 to 0.56, the sheet resistance decreased from 21.7 Ω/sq to 10.9 Ω/sq for the laser patterned 8 nm-thick Cu thin film, as shown in Fig. 2b. Compared with the as-deposited Cu film, the sheet resistance of the 8 nm-thick laser patterned Cu film increased as significant amount of metal was removed during laser patterning. However, the increase is still within a reasonable range. It should be noted that sheet resistance on the order of ~30 Ω/sq for a transparent conductor is comparable to ITO, a common transparent conducting oxide and suitable for use in optoelectronic devices.

Furthermore from Fig. 2 it can be found that the sheet resistance of 8 nm-thick laser patterned Cu film with different areal densities has a much larger standard deviation than that of the as-deposited Cu film. This indicates that the sheet resistances at different locations on the laser patterned Cu film are not as uniform as those on the as-deposited Cu film. This will potentially affect the current flow on the laser patterned surface and thus degrade the photoelectric performance of the laser fabricated film. The uniformity of the laser patterned hole array will be further investigated to resolve this issue.

### Visible transmittance

**Figure 3:**
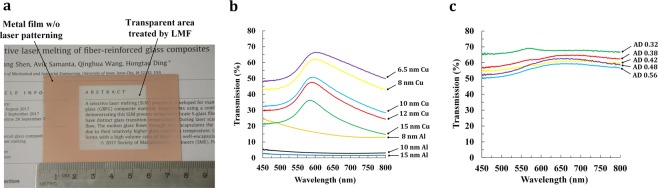
(**a**) A large LMF surface is placed on the text of a research paper illustrating its high visible transmittance. The size of the LMF surface is 36 mm × 36 mm; Visible transmittance of (**b**) as-deposited Cu and Al films as a function of film thickness and (**c**) 8 nm-thick laser patterned Cu thin film as a function of areal density (AD).

Optical transparency of the LMF-built sample is demonstrated by clear texts seen through a typical LMF-built sample area of 36 mm × 36 mm (Fig. 3a). The effects of various metal films and thickness were experimentally evaluated by performing transmittance measurements using a UV-Vis spectrometer (USB4000, Ocean Optics). It is generally believed that the ultra-thin metal film should be semi-transparent in order to achieve high visible transmittance after laser patterning^39^. In this work, Cu films with thicknesses of 15 nm, 12 nm, 10 nm, 8 nm and 6.5 nm were deposited on the glass substrate. The as-deposited Cu film with a thickness of 15 nm only has an average visible transmittance of 23.8% in the visible wavelength range of 450 nm to 800 nm, as shown in Fig. 3b. When the thickness of Cu film was reduced to 10 nm and 8 nm, the average visible transmittance increased to 37.7% and 50.1% respectively. With the thickness of Cu film further reduced to 6.5 nm, the average visible transmittance increased further to 55.7%. According to literature^63^, it is believed that the Cu film thickness can be further reduced to ensure that the as-deposited Cu film has an average visible transmittance of ~60% in the visible wavelength range of 450 nm to 800 nm with satisfying sheet resistance. However, the sheet resistance of the as-deposited Cu film with very low film thickness will be extraordinarily high, which will affect the electrical conductivity of laser patterned film. Thus, the film thickness should be properly selected in order to maintain balance between the sheet resistance and visible transmittance on the as-deposited film. In the meantime, the average visible transmittance of Al films with thicknesses of 8 nm, 10 nm and 15 nm deposited on the glass substrate is shown in Fig. 3b. The average visible transmittance of the 15 nm-thick Al film was 1.9%. When the thickness of Al film was reduced to 10 nm and 8 nm, the average visible transmittance increased to 3.7% and 17.4% respectively. It can be found the as-deposited Cu film has higher average visible transmittance than the as-deposited Al film with same film thickness. Combined with the sheet resistance results, we conclude that Cu film is a suitable material choice for LMF as it helps achieve better combined photoelectric properties on the LMF surface.

Areal density of laser patterning is a key parameter for the visible transmittance. As the areal density decreased from 0.56 to 0.32, the average visible transmittance increased from 56.2% to 67.2%, as shown in Fig. 3c. The average visible transmittance of the as-deposited 8 nm-thick Cu thin film is 50.1%. Laser patterning process increased the average visible transmittance by 17.1% using an areal density of 0.32. The LMF surface with the areal density of 0.32 also has a sheet resistance of 21.7 Ω/sq, demonstrating superior photoelectric properties.

This study has demonstrated the importance of metal film thickness for achieving satisfying visible transmittance and electrical conductivity, as they are contradicting properties for the laser patterned films. On the one hand, the visible transmittance of the as-deposited metal film decreases as the film thickness increases. On the other hand, the electrical conductivity increases as the film thickness increases. For the as-deposited Cu film, as the film thickness increased from 6.5 nm to 15 nm, the sheet resistance gradually decreased from 11.0 Ω/sq to 2.4 Ω/sq. Thus even though lower thickness of the as-deposited metal film is preferred for achieving a good optical transparency, low sheet resistance with a good amount of conducting material also needs to be considered.

The laser ablation process during laser patterning significantly affects the visible transmittance property. As nanosecond laser patterning is a thermal process that experiences melting phase transition, ablating the metal film with a thickness on the order of several micrometers will produce large amount of debris and result in high surface roughness due to the instability of the melt surface. In this work, ultrathin metal film with a thickness on the order of ~10 nm is patterned using the nanosecond laser which creates a relatively clean surface with low surface roughness. Still, from the optical micrograph of the patterned hole array as shown in Fig. 1b, it can be inferred that there are burrs generated around the hole circumference after LMF process. These burrs are attributed to the nanosecond laser fluence used in the experiments that generated distinct heat-affected zones. It can be seen in the figure that the laser power intensity caused ejection of condensed and solidified nanoparticle clusters which were deposited around laser patterned holes. These burrs can cause scattering of light and increase surface roughness^25^, which may result in poor optical and electrical performance. Slight aperiodicity of the laser patterned hole array and imperfect hole shape are also observed which could lead to electrical shorts. Laser parameters, pattern design and areal density of the laser patterned film can be further adjusted to achieve the photoelectrical performance that can balance the visible transmittance and electrical conductivity.

### THz bandpass filtering effect

We measured the THz transmission of different surfaces, including pure quartz and glass substrates, quartz and glass substrates coated with 8 nm-thick Cu film and laser patterned Cu films on the quartz substrates utilizing the 1000D TeraView THz-Time Domain Spectrometer.

**Figure 4:**
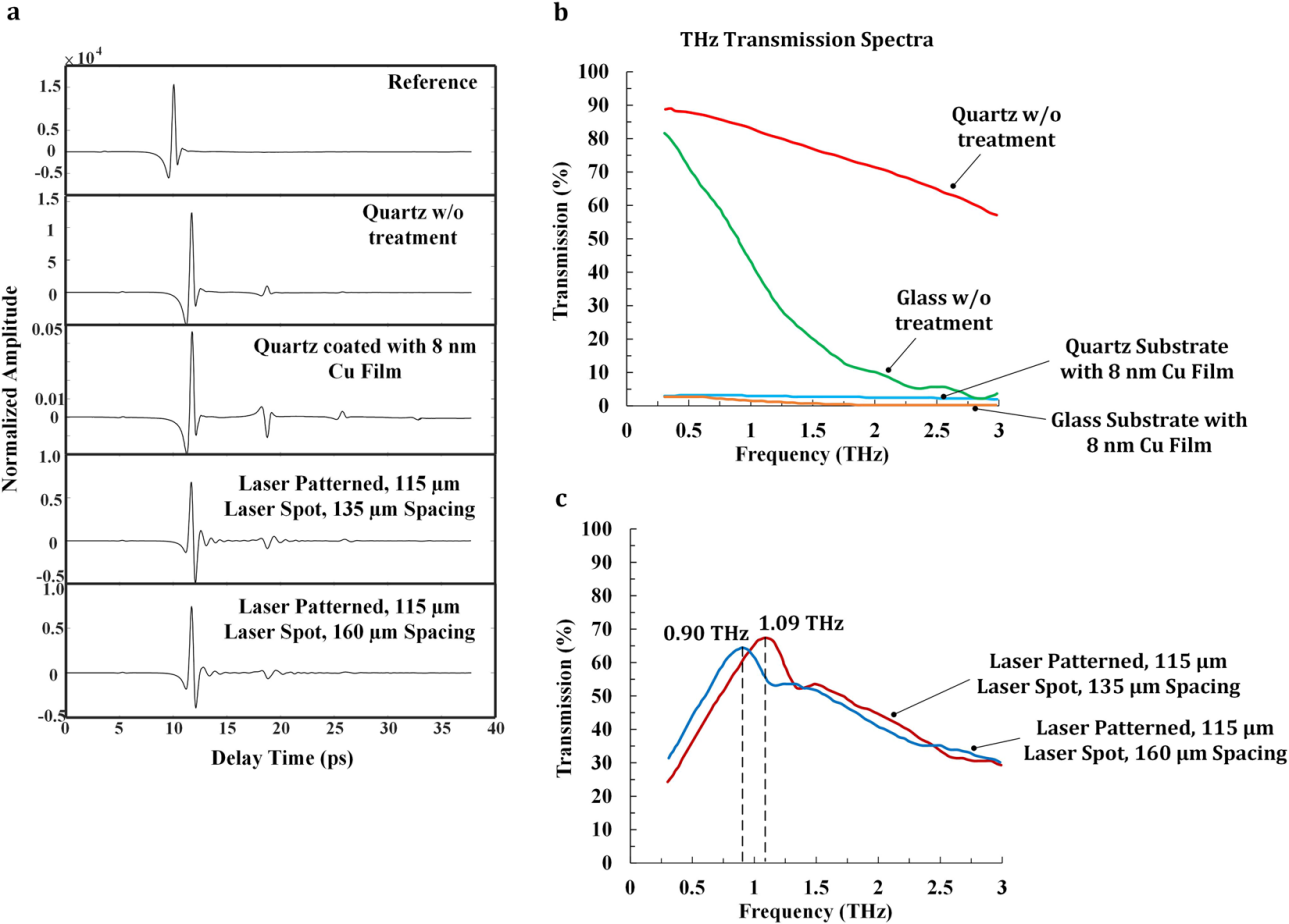
(**a**) Time domain data collected for air reference, pure quartz substrate, quartz substrate coated with 8 nm-thick Cu film, laser patterned surface with laser beam diameter of 115 µm and hole spacing of 135 µm and laser patterned surface with laser beam diameter of 115 µm and hole spacing of 160 µm. There is notable oscillations in the waveform of the laser patterned Cu films; (**b**) THz transmission spectra for quartz and glass substrates without treatment and quartz and glass substrates coated with 8 nm-thick Cu film; (**c**) THz transmission spectra for laser patterned surface with laser beam diameter of 115 µm and hole spacing of 135 µm and laser patterned surface with laser beam diameter of 115 µm and hole spacing of 160 µm.

The time-domain waveforms corresponding to the transmitted THz pulses through different interfaces are shown in Fig. 4a. The temporal scan window was set large enough in order to accurately obtain the linewidths of the resonance features. By comparing the waveforms of air reference, pure quartz substrate, and quartz substrate coated with 8 nm-thick Cu film, notable different features can be found in these time-domain traces. First an apparent time delay can be found for the bipolar pulse feature in the waveform of pure quartz substrate compared with that of air reference because of the difference in their refractive indexes. Second, the air reference waveform exhibits an almost one cycle pulse which corresponds to the incident THz wave. For the waveforms of pure quartz substrate and quartz substrate coated with 8 nm-thick Cu film, there are several etalons after bipolar pulse. In comparison with the waveforms of pure quartz substrate and quartz substrate coated with 8 nm-thick Cu film, it appears that there are damped oscillatory waveform features present in the waveforms for laser patterned Cu film and the spectral features of the transmission resonances are contained in these oscillations. These oscillations can be attributed to the resonant interaction of the THz pulse with the periodic surface structure patterned on Cu film. In general, the magnitude of the oscillations corresponds to the magnitude of the resonance feature, while the oscillation duration corresponds to the linewidth of that feature. From Fig. 4, it is apparent that the bipolar pulse feature in the waveforms of laser patterned Cu film shows a sign reversal relative to the waveform of pure quartz substrate. The sign reversal can be potentially attributed to the significant reshaping due to diffraction caused by the interaction between the incident THz pulse and laser patterned periodic hole array structure^26^. As a result of this reshaping process, the transmitted THz pulse might exhibit an additional $\pm \,\frac{\pi }{2}$ phase shift in the low frequency limit. Thus the sign reversal of the transmitted time domain waveform would correspond to the phase shift imparted to a THz pulse transmitted through the laser patterned periodic hole array structure^21,26^.

The THz transmission spectra of the pure quartz and glass substrates, the quartz and glass substrates coated with 8 nm-thick Cu film, and the laser patterned Cu films on quartz substrate were collected by THz-Time Domain Spectroscopy (THz-TDS) technique. Experimental results indicate that the pure quartz substrate has an average THz transmission of 75% in the frequency range of 0.3~3 THz. However, the pure glass substrate only has an average THz transmission of 33% in the frequency range of 0.3~3 THz and its THz transmission intensity profile exhibits a significant drop in this frequency range. After 8 nm-thick Cu film was deposited on the pure quartz and glass substrates, the average THz transmission was decreased to 3% in the frequency range of 0.3~3 THz for both of the substrates, which can be found in Fig. 4b. To achieve THz filtering effect, it is required that the THz transmission profiles between the pure quartz substrate and 8 nm-thick Cu film coated on quartz substrate should have significant difference. The reason could be attributed to that if no difference can be found between these two transmission profiles, no transmission peak can be generated after laser patterning. Therefore our research shows that quartz is more suitable as the substrate required for achieving THz filtering effect. Thus in order to achieve the best combined properties of high transmittance, low sheet resistance and THz filtering effect, quartz and Cu are selected as the substrate material and metal film material respectively.

This work for the first time demonstrated ultra-thin metal film with a thickness on the order of ~10 nm to efficiently block the THz wave. Although the AC complex conductivity of metals at THz frequencies is well known, the skin depths of metals reported in literature were on the order of at least 100 nm^64–67^. The thickness of the metal film used in this work is on the order of ~10 nm, which is one magnitude lower and significantly different than those reported in literature. Based on the research work of Cao and Nahata^21^, they have proposed to examine the properties of the wave field propagating on and through the pure metal film in terms of the properties of SPPs. They concluded that because of failing to obey the conservation of energy and momentum laws, electromagnetic radiation incident on a plane metal-dielectric interface cannot couple to the SPP waves. Therefore, it can be inferred that conservation of energy and momentum laws are still not obeyed with the metal film thickness on the order of ~10 nm. Future work will be focused on more investigation to elucidate the fundamentals of this interesting phenomenon.

For the LMF experiments, two different sets of experimental parameters were used for laser patterning of Cu film: Pattern 1 used a laser beam diameter of 115 µm, a hole spacing of 135 µm and an areal density of 0.43; Pattern 2 used a laser beam diameter of 115 µm, a hole spacing of 160 µm and an areal density of 0.59. After LMF process, the LMF surfaces exhibited clear THz filtering effect with specific resonance frequency and the resonance frequency could be tuned by changing the experimental parameters, as shown in Fig. 4c. Since the Cu film was deposited on the quartz substrate, there are three interfaces surrounded this stacked structure: metal-air interface, quartz-air interface and metal-quartz interface. The metal-air interface and quartz-air interface were ignored when calculating the location of the resonant transmission peaks. The quartz substrate causes a downward shift in resonant frequency, which reaches a limiting value scaled by the square root of the mean dielectric constant^68^. The equation that is used to calculate the approximate locations of the resonant transmission peaks is defined as Equation 1 ^21,26,69^:

1 $${\lambda }_{peak}=\frac{P}{\sqrt{{i}^{2}+{j}^{2}}}\sqrt{\varepsilon }$$

where *P* is the physical periodicity, ε = 3.7~4.2 is the dielectric constant of the interfacial dielectric media for metal-quartz interface, and *i* and *j* are indices corresponding to the resonance order.

Using the above equation for calculation of the resonant transmission peaks and conversion between wavelength and frequency, the approximate locations of the resonant transmission peaks for both experimental conditions will be: 1.08~1.16 THz (wavelength of 259.7~276.7 µm) for Pattern 1 and 0.91~0.97 THz (wavelength of 307.8~327.9 µm) for Pattern 2. The experimental results indicate that the resonant transmission peak occurs at 1.09 THz (wavelength of 274.5 µm) for Pattern 1 and 0.90 THz (wavelength of 333.1 µm) for Pattern 2, as shown in Fig. 4c. The experimental results and theoretical calculations exhibit good agreement.

The experimental results show a secondary resonant transmission peak and a rather wide FWHM bandwidth, which is caused due to the following laser patterning defects. Firstly, laser patterned hole array exhibited distinct aperiodic nature, as can be found in Fig. 1b. The imperfect periodicity of the laser pattered hole array was caused by the manufacturing inaccuracy during LMF process. Secondly it can be found that the geometry of the laser patterned spot is not perfectly circular. Thirdly, there are burrs generated around the hole circumference after LMF process, as discussed in the previous section. These burrs exhibit distinct heat-affected zones and will cause scattering effect and increase surface roughness. All of the above-mentioned factors contribute to the occurrence of the secondary peak and a relatively wide FWHM bandwidth of Δf ≈ 2 THz, which is higher than the ideal FWHM bandwidth of 0.2~0.3 THz^70,71^. By using a laser that generates high-quality Gaussian beam, the above issues are expected to be resolved, and thus the secondary resonant transmission peak can be removed and the FWHM bandwidth of the LMF surface can be much narrower, which will facilitate the design and fabrication of narrow-band THz filter.

Table 1 shows the combined photoelectric properties for LMF surfaces for Pattern 1 and Pattern 2. Both visible transmittance and sheet resistance were measured for more than three times at various locations on a specimen and the average value with standard deviation was reported. It can be found that the two surfaces exhibit a visible transmittance of ~60%, a sheet resistance of ~20–30 Ω/sq and a specific resonance frequency with ~2 THz FWHM bandwidth. The reason that the visible transmittance of the LMF surfaces fabricated on the quartz substrate is lower than that on the glass substrate can be attributed to the following reasons: (1) The visible transmittance of quartz substrate is slightly lower than that of the glass substrate; (2) The thickness of the as-deposited films on different substrates is slightly different. In addition, it is noted that the visible transmittance of Pattern 2 is larger than that of Pattern 1, and the sheet resistance of Pattern 2 is higher than that of Pattern 1 even though the areal density of Pattern 2 is larger than that of Pattern 1. This variation was mainly attributed to the various Cu film thickness employed in Pattern 1 and Pattern 2. The visible transmittance of the as-deposited Cu film of Pattern 2 was around 4% higher than that of Pattern 1, while the sheet resistance of the as-deposited Cu film of Pattern 2 was 3.4 Ω/sq higher than that of Pattern 1. Therefore, although the combined photoelectric properties need further refinement, we demonstrate that the LMF method has potential to be used for the large scale and low-cost fabrication of the multi-functional THz components.

**Table 1:** Combined photoelectric properties for Pattern 1 and Pattern 2.

	Visible transmittance	Sheet resistance	Resonance frequency	FWHM width
Pattern 1	56.9 ± 0.4%	16.3 ± 1.8 Ω/sq	1.09 THz	1.94 THz
Pattern 2	57.2 ± 0.6%	31.9 ± 3.2 Ω/sq	0.90 THz	2.29 THz

From the THz spectra of two patterns, it is found that the peak transmission intensity of Pattern 1 is 67.4%, which is slightly higher than the peak intensity of 64.5% for Pattern 2. This could also be attributed to the slight difference between the thicknesses of Cu films used for the two patterns. The Cu film for Pattern 1 is slightly thicker than that of Pattern 2. As a result, a higher peak transmission intensity is observed for the THz spectrum of Pattern 1 rather than Pattern 2. This phenomenon indicates that metal thickness is important for efficient THz transmission through laser patterned metal film. With higher metal film thickness, higher THz transmission intensity is facilitated. In addition, the electrical conductivity will be higher while the visible transmittance will be lower with higher film thickness, as discussed in the previous section. To balance the three properties, proper metal film thickness should be used in the LMF experiments. It is also found that the locations of resonant transmission peaks for Pattern 1 and Pattern 2 are different since different areal densities were used for these two patterns during LMF experiments. The experimental results indicate that areal density affects the THz transmission of the LMF surface. Literature work indicates that the physically relevant parameter that determines extraordinary THz transmission properties is the hole size for a fixed hole periodicity^31^. Therefore, metal film thickness, hole size, and areal density are key parameters that determines the combined performance of visible transmittance, electrical conductivity and THz filtering effect. According to the specific requirements of any electronic devices, these parameters could be further optimized to achieve the desired functions.

### Processing efficiency

The LMF process developed in this work is highly time-efficient and cost-effective as it only involves two simple steps for the three integrated functionalities. The other state-of-art nanofabrication methods usually involve many steps, which are time-consuming and costly for large area processing. For example, photolithography method^27–29^ has been applied for fabricating THz transmission subwavelength hole arrays, which involves at least six steps including ultrasonic cleaning, spin coating, soft-bake, UV exposure, chemical development and reactive ion etching. The production time for such a metasurface with metallic hole array is at least one magnitude longer than the LMF process.

One key advantage of the LMF process is that it utilized a laser beam diameter on the order of several hundreds of µm during laser scanning of the thin metal film. Thus the processing efficiency has been significantly increased compared with the existing research work done by other type of nanosecond laser^39^, picosecond laser^72^ or femtosecond laser^73^, which used a laser beam diameter on the order of only several µm. It is expected that the laser beam diameter could be further increased to several millimeters while maintaining the similar laser fluence to further enhance the processing efficiency. Furthermore it should be noted that the LMF process just used an affordable nanosecond laser with a repetition rate of 10 Hz and thus currently it took around one hour to fabricate the transparent conducting film with an area of 36 cm × 36 cm using the optimal laser processing parameters. The processing speed can be significantly further optimized by using lasers with higher repetition rate. If a laser at 10 kHz pulse was used, the processing time could be reduced to 6 seconds. It should also be noticed that even with such high laser fluence, no structural deformation and damage were found on the quartz substrate. All of the above-mentioned advantages of this LMF process will render more practical treatment of glass-based materials to produce multi-functional THz components for various applications.

### Understanding the LMF THz bandpass filtering via numerical simulations

**Figure 5:**
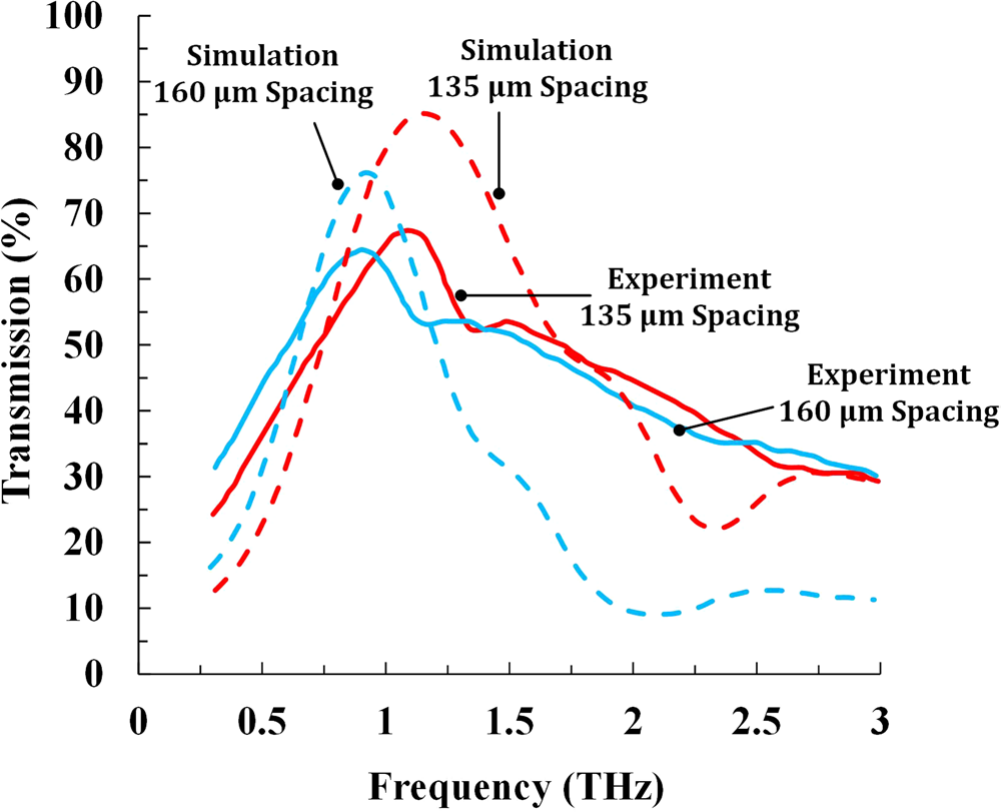
Experimental and simulated THz transmission of laser patterned surface with laser beam diameter of 115 µm and hole spacing of 135 µm and laser patterned surface with laser beam diameter of 115 µm and hole spacing of 160 µm.

Finite element method model was developed utilizing COMSOL Wave Optics module^74^ to help determine the fundamental mechanism of THz bandpass filtering for the LMF-built samples. Figure 5 shows the simulated THz transmission of the LMF surfaces created with identical laser beam diameter of 115 µm, and different hole spacings of 135 µm with an areal density of 0.43 and 160 µm with an areal density of 0.59. The simulations were set up with periodic boundary conditions assuming a unit cell of one hole with spacing defined by the LMF process. The source and listener ports were set in the simulation to generate and measure the transmitted THz wave through the patterned Cu film, respectively. The simulations were performed utilizing the refractive index of Cu in THz domain obtained from literature^75^. For the case with the hole spacing of 135 µm, the resonance frequency of this structure can be found at around 1.09 THz. While for the case with the hole spacing of 160 µm, it exhibits a red-shifted resonance frequency at 0.90 THz compared to the case with the hole spacing of 135 µm. The red shift feature is attributed to the larger hole spacing which results in a larger wavelength value for the location of the resonant transmission peak, as indicated in Equation 1.

Table 2 presents the error of the resonance frequency and resonant transmission intensity between the experimental and simulation results. It is found that the error of the resonant frequency between the experimental and simulation results is less than 6%, while the error of the resonant transmission intensity between the experimental and simulation results is a little higher with a value of 15~21%. This indicates that the simulated results agree well the experimental results in terms of the resonance location. However, there is discrepancy for the resonant transmission intensity between the experimental and simulated results which could be attributed to the imperfect nature of the laser patterned hole array caused by the manufacturing inaccuracy during laser micro-machining, as discussed in the previous section. The processing parameters during numerical simulation were assumed to be ideal while experimental error occurred for LMF experiments. Simulation results indicate that hole spacing and hole geometry are important parameters that will affect the quality of THz transmission and these two parameters should be further optimized. In addition, if an industrial laser with high repetition rate and low pulse energy can be used, there will be less metal deposition around the laser ablated holes and thus the surface roughness of the LMF surface can be reduced, which will eliminate the scattering effect and enhance THz transmission through the LMF surface^25^. Although the discrepancy exists, the good agreement between the experimental and simulation results indicates that LMF process is a promising method for fabrication of transparent conducting THz bandpass filters.

**Table 2:** Error of the resonance frequency and resonant transmission intensity between the experimental and simulation results.

Hole spacing	Resonance frequency (THz)			Resonant transmission intensity (%)		
	Simulation	Experimental	Error	Simulation	Experimental	Error
135 µm	1.15	1.09	5.5%	85.2	67.4	20.9%
160 µm	0.92	0.90	2.2%	76.2	64.5	15.4%

## Conclusions

This work developed an innovative Laser-based Metamaterial Fabrication (LMF) process to enable time-efficient and cost-effective fabrication of transparent conducting THz bandpass filter. The laser patterned ultra-thin metal film exhibits combined surface properties of high visible transmittance, good electrical conductivity and THz bandpass filtering effect. This LMF process proves the feasibility that these three important functionalities can be integrated into one single surface. This process significantly improves the processing efficiency and reduces production cost compared with the existing laser surface patterning methods for fabrication of functional photoelectric surfaces. By using a high-energy nanosecond laser, it renders practical treatment of macroscale transparent substrates for various military, industrial and transportation applications.

More future efforts will be devoted for the development of tuning methods for the THz bandpass filter, such as electrical, magnetic, and mechanical methods. The developed THz bandpass filters with a frequency-domain modulating functionality will be tested and used for various THz applications, such THz gas-phase spectroscopy^76,77^ and THz imaging^78^.

## Methods

### Optical micrograph

The optical micrograph of the laser patterned hole array structure was captured using a measuring optical microscope.

### Sheet resistance measurement

**Figure 6:**
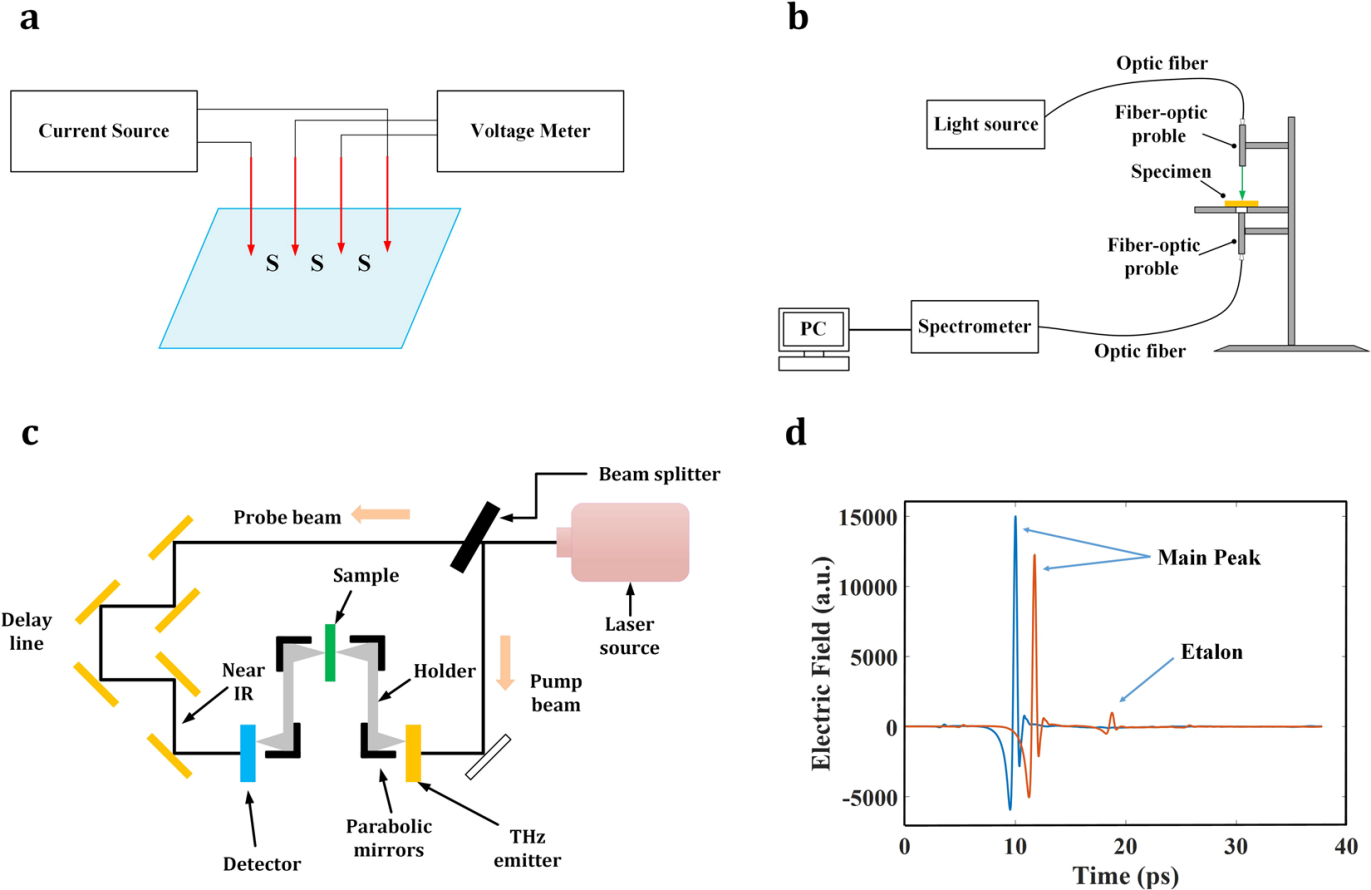
Experimental setup for (**a**) sheet resistance measurement; (**b**) visible transmittance measurement; (**c**) THz time domain spectroscopy; and (**d**) Time-domain spectrum collected by THz-TDS.

The sheet resistance of the laser patterned hole array were tested with a digital four point probe sheet resistivity measurement system (Signatone Pro4 series), which is connected to a sourcemeter (Keithley 2400 series) for sheet resistance value reading. The schematic for sheet resistance measurement is as shown in Fig. 6a. Four metallic probe pins are applied to the surface of a specimen, being lined up, and current is made to flow through the two outer most probe pins. When the difference in potential between the two intermediate probe pins is measured, the sheet resistance can be found from Equation 2:

2 $$\rho s=\frac{V}{I}\frac{\pi }{ln2}=4.5325\frac{V}{I}$$

where *ρs* is the sheet resistance with the unit of Ω·sq, *V* is the voltage between the inner probes, and *I* is the current through the outer probes. *S* is the needle spacing as shown in the schematic. Each specimen surface was measured for three times at various locations, and the averaged sheet resistance was obtained.

### Optical transmittance measurement

The optical transmittance of laser patterned microhole array was measured using a UV-Vis spectrometer (USB4000, Ocean Optics Co.) with normal incidence. The schematic for optical transmittance measurement is as shown in Fig. 6b. The UV-Vis spectrometer measures the transmittance of the specimen surface in the wavelength range of 400~1000 nm. Before transmittance measurement, calibration of the transmittance scale was performed by measuring the transmittance through air. Then the specimen was placed on the optical path of normal incidence for the actual transmittance measurement. During the transmittance measurement, light from a visible and near-infrared light source (HL-2000; Ocean Optics Co.) was fed through the illuminating fiber-optic probe, directed through the specimen placed on top of the pinhole, and into the quartz fiber-optic probe coupled to a USB4000 spectrometer. OceanView® software was utilized to process and visualize the transmittance measurement results. Each specimen surface was measured for three times at various locations, and the averaged spectral transmittance was obtained.

### THz-Time domain spectroscopy

Experimental THz transmission spectra, were generated using THz-Time Domain Spectroscopy technique^79,80^. THz-TDS is conducted by generating a coherent pulse of EM radiation at THz frequencies, passing the radiation through the sample of interest, and detecting the transmitted radiation in a time dependent manner. Figure 6c shows a general schematic of a typical THz-Time Domain Spectrometer. The THz-Time Domain Spectrometer is comprised of three main elements: a near infrared laser, a THz emitter, and a time domain detection system.

A 1000D TeraView spectrometer (TeraView Limited, Cambridge, UK) was used to collect the THz-TDS data. This instrument utilizes a Ti:Sapphire (Ti:Al_2_O_3_) near infrared laser to generate a femtosecond laser pulse over a period less than 100 fs at a wavelength of 800 nm. This pulse is then split into a probe and pump beam. The pump beam strikes a polarized, low temperature grown GaAs semiconductor, quickly creating electron-hole pairs to generate a broadband of electromagnetic radiation. The broadband nature of this radiation makes it well suited for solid-phase measurements where spectral bands are relatively broad compared to gas-phase spectra.

Time information is collected using a delay line. The probe beam is directed to the delay line, where the delay line position allows for sampling of the time domain signal in a discrete and time-integrated manner. The 1000D TeraView spectrometer is equipped with a photoconductive (PC) detector. This detector operates by utilizing low temperature grown GaAS semiconductor (band gap ~1.5 eV at STP). After the pump beam passes through the sample compartment and the probe beam passes through the delay line, they are directed to illuminate the detector. The higher energy probe beam generates electron-hole pairs in the semiconducting PC material, while the mobile electrons are accelerated by the oscillating electric field vector of the THz radiation, creating a measurable current between the antenna electrodes of the detector assembly. The semiconducting material used in the Teraview 1000D spectrometer is GaAs with 1.5 eV band gap at room temperature^79,80^.

Each THz-TDS spectrum was collected as 1800 co-added scans attained over one minute. Three air-reference spectra and nine sample spectra were collected for each quartz sample. Confounding water vapor lines were avoided by purging the sample compartment with dried air. Time-domain spectra were processed subsequently to achieve frequency-domain absorption spectra. Each time-domain spectrum was truncated just before the first etalon and the truncated time-domain spectrum data was zero-filled to 8192 (2^13^) points as shown in Fig. 6d. A boxcar apodization function was applied prior to the fast Fourier transform to yield the corresponding frequency-domain electric field spectrum. Transmission spectra were then calculated as the ratio of the sample to air electric field spectra in the frequency domain. The resolution of the resulting spectra was 0.036 THz over a spectral range of 0.3~3 THz.

### Finite element method (FEM) simulations

To better explain and verify the experimental results, THz transmission of the LMF surface was simulated using the Wave Optics Module of the finite element method (FEM) software COMSOL Multiphysics®^81^. The simulation domain of the structure was reduced to a single unit cell with periodic boundary conditions, to simplify the computation. Two ports, source and listener ports were placed on the interior boundaries of this unit cell to determine the transmission characteristics using the S-parameter calculation. The hole diameter and spacing were varied in the simulation corresponding to actual experimental conditions to determine the impact of these parameters on the THz transmission of the patterned Cu films. The geometry, material and boundary conditions for the FEM simulation of the THz transmission through the LMF surface will be discussed in this section.

**Figure 7:**
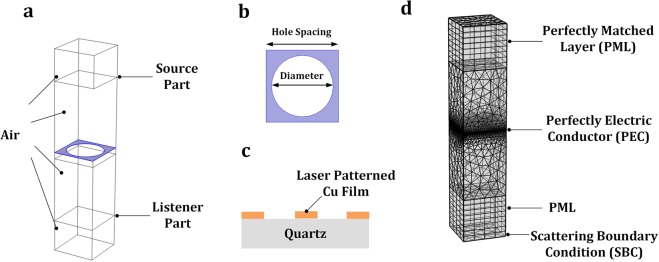
3D simulation setup of the LMF surface (**a**) the whole simulation domain; (**b**) unit cell structure of the LMF surface; (**c**) material setup of the LMF surface; and (**d**) mesh setup and boundary conditions for the simulated LMF surface with the maximum boundary element size set equal to λ/6.

In the model, the simulated surface structure is consisted of a unit cell inside the periodic micro-hole array patterned on the quartz substrate coated with ultra-thin Cu film, as shown in Fig. 7a. The thicknesses of the Cu film and the quartz substrate, the laser spot diameter and the laser patterned hole spacings were set corresponding to the experimental conditions in LMF experiments. The optical properties of the Cu film is frequency dependent in the THz range 0.3~3 THz, and the refractive index for this material was extracted from the experimental data of Sun *et al*.^75^. The substrate material used in this work was defined as quartz, and the refractive index of this material was obtained from the built-in library of COMSOL. The rest of the simulation regions are air-filled, and the refractive index in these regions was also obtained from the built-in library of COMSOL. The simulated surface structure and material setup of the simulation domain are shown in Fig. 7b,c.

The THz wave propagated in a hypothetical air-filled top region was set with perfect electric conductors (PEC) on the side normal to the wave propagation direction while the bottom side was set as scattering boundary condition (SBC). The simulation domain of the LMF surface was reduced to a single unit cell with the Floquet-periodic boundary conditions applied on four sides of the cell to simulate the infinite 2D array. The air-filled top and bottom regions were set to perfectly matched layers (PMLS) for absorbing the excited mode from the source port and any higher order modes generated by the periodic structures. The structure mesh is physics-controlled with the maximum element size equal to $\frac{\lambda }{6}$ with *λ* defined as the wavelength of the incident wave. The paired boundaries of the periodic boundary regions were set with the identical surface meshes. The mesh setup of the LMF surface is shown in Fig. 7d.
